# Willingness to pay for hepatitis B vaccination in Selangor, Malaysia: A cross-sectional household survey

**DOI:** 10.1371/journal.pone.0215125

**Published:** 2019-04-09

**Authors:** Yogambigai Rajamoorthy, Alias Radam, Niazlin Mohd Taib, Khalid Ab Rahim, Subramaniam Munusamy, Abram Luther Wagner, Mudatsir Mudatsir, Abdullatif Bazrbachi, Harapan Harapan

**Affiliations:** 1 Department of Economics, Faculty of Accountancy and Management, Universiti Tunku Abdul Rahman, Selangor, Malaysia; 2 Department of Economics, Faculty of Economics and Management, Universiti Putra Malaysia, Selangor, Malaysia; 3 Department of Medical Microbiology and Parasitology, Faculty of Medicine and Health Sciences, Universiti Putra Malaysia, Selangor, Malaysia; 4 Centre for Language and Foundation Studies, Manipal International University, Negeri Sembilan, Malaysia; 5 Department of Epidemiology, University of Michigan, Ann Arbor, Michigan, United States of America; 6 Department of Microbiology, School of Medicine, Universitas Syiah Kuala, Banda Aceh, Indonesia; 7 Medical Research Unit, School of Medicine, Universitas Syiah Kuala, Banda Aceh, Indonesia; 8 School of Biomedical Sciences, University of Western Australia, Nedlands, Western Australia, Australia; Children's Mercy Hospitals and Clinics Department of Pathology and Laboratory Medicine, UNITED STATES

## Abstract

**Background:**

In Malaysia, one million individuals are estimated to be infected with the hepatitis B virus. A vaccine for infants has been compulsory since 1989, whereas those born before 1989 need to spend their own money to be vaccinated in private clinics or hospitals. The aim of this study was to investigate and ascertain the determinants of willingness to pay (WTP) for adult hepatitis B vaccine in Selangor, Malaysia.

**Methods:**

In 2016, 728 households were selected through a stratified, two stage cluster sample and interviewed. Willingness to pay for hepatitis B vaccine was estimated using the Contingent Valuation Method, and factors affecting WTP were modelled with logit regression.

**Results:**

We found that 273 (37.5%) of the households were willing to pay for hepatitis B vaccination. The mean and median of WTP was estimated at Ringgit Malaysia (RM)303 (approximately US$73) for the three dose series. The estimated WTP was significantly greater in those with higher levels of education, among Malays and Chinese (compared to others, predominantly Indians), and for those with greater perceived susceptibility to hepatitis B virus infection. Other factors–perceived severity, barriers, benefits and cues to action–were not significantly associated with WTP for adult hepatitis B vaccination.

**Conclusion:**

Additional resources are needed to cover the households that are not willing to pay for hepatitis B vaccination. More awareness (particularly in regards to hepatitis B virus susceptibility) could change the national perception towards self-paid hepatitis B virus vaccination and increase hepatitis B vaccine coverage.

## Introduction

The World Health Organisation has estimated that, worldwide, 257 million people are living with hepatitis B virus (HBV). Chronic infection can lead to HBV-related liver cirrhosis or hepatocellular carcinoma, which resulted in 887,000 deaths in 2015 [1]. Progression to chronic hepatitis B (HepB) is more pronounced when infants acquire HBV (with 80%-90% likelihood of chronic infection), compared to adults (with 5%-10% likelihood of chronic infection) [2, 3]. Nonimmune adults who are acutely infected could be important sources of HBV transmission.

The burden of disease due to HBV is among the highest of any vaccine-preventable infection within the country. In Malaysia, whose population is 31.9 million, 6.5% are positive for HBV surface antigen (HBsAg) and 51% are positive for HBsAg antibody (HBsAb) [4]. One million individuals are estimated to be chronically infected with HBV [4], corresponding to a prevalence of >5% [5]. Chronic HepB accounts for >80% of hepatocellular carcinoma cases reported in Malaysia [4]. The government estimates that incidence of HepB has increased from 2.26/100,000 population in 2010 to 12.94/100,000 population in 2014 [6, 7], and the incidence and number of HepB cases in Malaysia is projected to increase through 2030 [8]. From these figures, Malaysia is considered to be a country with intermediate-high levels of HBV endemicity [5], and acute and chronic complications from the virus result in an enormous public health and health system problem in Malaysia.

Because chronic liver disease develops over years and contributes to direct and indirect medical costs, its economic impact affects both lost work wages and loss of long-term productivity [9]. A study conducted in South Korea estimated that the total indirect and direct cost of HBV-related disease totalled US$959.7 million, equivalent to 3.2% of all health expenditures in South Korea [10]. The large costs of HBV infection necessitate a discussion of the merits of an adult HepB vaccination program.

In Malaysia, individuals born before 1989 are not covered under the compulsory HepB vaccination programme. Currently, adult vaccinations are only given to high-risk groups, such as healthcare workers in public clinics and hospitals. Most HepB studies in Malaysia concern health care workers and medical graduates [11–13]. Most adults in Malaysia must actively decide to immunise themselves against the HBV. Ng *et al*. [14] have proposed initiating a voluntary vaccination program in Malaysia to prevent HBV. However, missing from this literature is an empirical study on willingness to pay (WTP) for HepB vaccine. These findings could guide strategies for pricing vaccines and programs for promoting vaccine uptake. We use the Health Belief Model (HBM) as a framework for identifying attitudinal predictors of WTP. The HBM is widely used, including in previous studies on HepB vaccination [15–18], and its components–perceived susceptibility to HepB, perceived severity of HepB disease, perceived benefits of HepB vaccination, perceived barriers in preventing HepB and cues to action for HepB vaccination–could be targeted for educational or informational interventions. Given the lack of information on adult perceptions of HepB and their WTP for a preventive intervention, the objective of this study was to discover households’ WTP for HepB vaccination, and to identify its sociodemographic and behavioural predictors.

## Methods

### Ethics approval

The study protocol was approved by the Institutional Review Board of Universiti Putra Malaysia, Selangor, Malaysia (UPM/FEP/TDPS/GS32435). All participants signed written informed consent forms prior to enrolment. Participation in this study was voluntary and no financial incentive was given. The work was carried out in accordance with The Code of Ethics of the World Medical Association (Declaration of Helsinki) for experiments involving humans.

### Study site, study design and sampling procedure

A cross-sectional household survey to determine the WTP and its predictors using Contingent Valuation Method (CVM) was conducted in nine districts of Selangor state, Malaysia, from January to May 2016. Selangor’s population of 5.79 million population makes it the most populous state and its ethnic diversity (56.9% Malay, 28.5% Chinese, and 13.5% Indian) roughly mirrors the country as a whole [19]. This study was part of hepatitis B in Malaysia Project and other aspects of the project have been published elsewhere [18, 20].

Mitchell and Carson [21] stated that a CVM study needs a large sample size to estimate the mean WTP to overcome problem of biases. Three main criteria are used to determine the sample size: (a) the deviation of the expected or acceptable the estimated WTP from the true WTP (Δ); (b) the relative error of the true WTP (V); and (c) precision. Using these three criteria and a formula suggested previously [21], the minimum sample size required was 683. This is based on the assumption that the deviation of the estimated WTP from the true value (Δ) was 15%, the relative error of the true WTP (V) was 2.0, the margin of error was 5% and the confidence interval was 95%.

A two-stage cluster sampling design with proportional allocation was used to obtain a representative sample. The sampling procedure was assisted by the Malaysia Department of Statistics. Briefly, Selangor state was divided into small areas known as enumeration block (EB). Each EB, consisting of between 80 and 120 living quarters (LQ), was clustered into four strata based on age. Out of 16,562 EBs for selected districts, 64 EBs were selected and within each EB, 12 LQs were selected randomly for a total of 768 LQs. In each LQ, one adult aged ≥20 years who was a Malaysian citizen was invited to participate in the study.

### Study instruments

The questionnaire used in this study included questions on sociodemographic characteristics, perceptions about HepB vaccine and WTP. Items on sociodemographic included age, gender, ethnicity, religion, marital status, education level, employment type and household monthly income. The HBM assessed respondents’ perception towards the HepB vaccination using questionnaires that have been published elsewhere [16, 22–24]. The number of questions for each domain as follows: perceived susceptibility to HepB (3 items) [16, 22], perceived severity of HepB disease (4 items) [16], perceived benefits of HepB vaccination (5 items) [16, 23], perceived barriers (3 items) [16, 24] and cues to action for HepB vaccination (3 items) [16]. The English version questionnaire was developed based on the existing literature and translated to the Malay language. The detailed questionnaire used in this study is given in S1 File. A panel consisting of a medical microbiologist, a public health doctor and internist were appointed to evaluate the content validity of the questionnaire in both versions. The finalised questionnaire was tested in a pilot study of 121 respondents selected via a convenience sample in a public place.

### Data collection

Face-to-face interviews in the respondent’s house were conducted in Malay or English by ten collection team members. All the members were second and third year university students, recruited from Universiti Tunku Abdul Rahman, Selangor, Malaysia. A short-course training programme was conducted for the interviewers. A major part of the training was on reducing five major types of CVM biases: strategic bias, starting point, yea-saying bias, hypothetical bias, and the information bias. Each of these biases was considered during questionnaire construction, as well as during the data collection. Efforts have been made to deal with each bias following previous recommendations [25–30]. For example, to avoid social desirability bias, the correct answers to the survey questions were not provided to interviewers. Then their interview skill was assessed in a pilot test where each of the interviewer was assigned to complete ten interviews. Additional training was conducted for some interviewers before the actual study.

Prior to the interview, an overview of the study aims was explained to potential participants and they were informed that could leave the study at any time. Those who agreed to participate were asked to sign an informed consent form. Participants were provided information on HBV infection (seriousness, current epidemiological situation, potential complications and prevention methods) using brochures from Ministry of Health of Malaysia.

### Measures

#### Dependent variable

The dependent variable in this study, WTP for HepB vaccination, was assessed using a CVM strategy. CVM is a stated preference technique whereby the bid has an unspecified probability distribution due to uncertain preferences based on an individual’s socioeconomic status [31]. Past literature using CMV has used different distributions in the bid amount [32–34]. In our study, a single-bounded closed-ended dichotomous choice question was used to estimate how much respondents were willing to pay for the three-dose HepB vaccine series. This strategy is the most commonly used method in environmental valuation because of its proposed incentive-compatibility properties [35] and because it is simple to estimate the WTP [36]. Although a double-bounded dichotomous choice strategy is statistically efficient compared to a single-bounded strategy [37], the double-bounded strategy has several disadvantages such as not being incentive-compatible in a hypothetical context [35], responses to first and second dichotomous questions may not be consistent [37] and it may suffer from a starting point bias [38, 39]. In addition, a single-bounded dichotomous choice question has some attractive features, is easier to implement and can avoid systematic bias or anchoring effect in responses [40].

**Fig 1:**
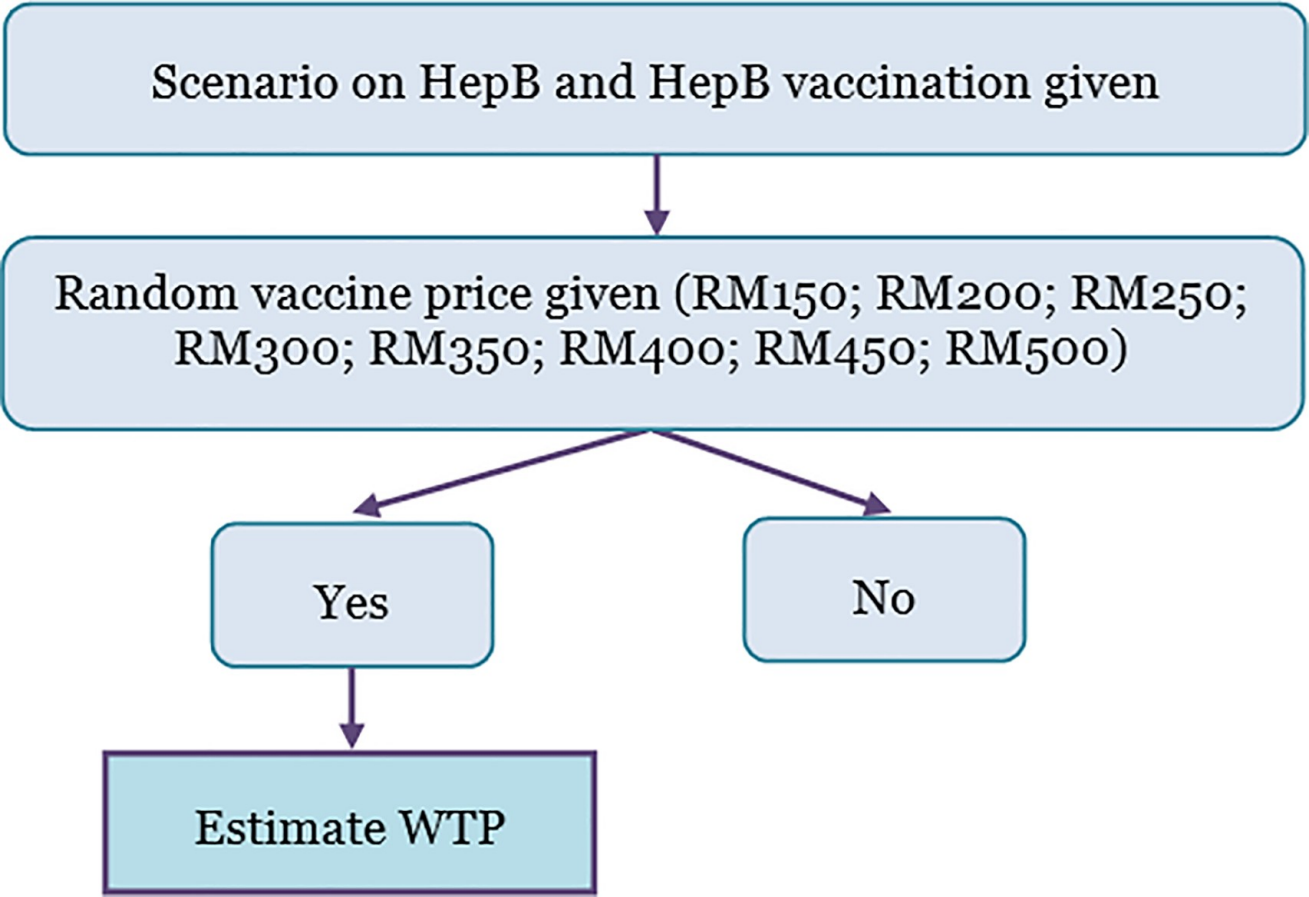
Flowchart how willingness to pay for hepatitis B vaccination was measured during the survey.

At the time of the survey, the prevailing market price for HepB vaccination in Malaysian Ringgit (RM) was around 60 (approximately US$14 using a November 2017 exchange rate) to 100 (US$24) for one dose. However, respondents were not informed about the market price; instead, they were asked according to a randomly chosen bid amount. Respondents were given a scenario where 30% of HBV-infected individuals faced a high chance of liver cancer, HepB vaccinations required three doses, the vaccine prevents HBV infection, and the Malaysian government provides free vaccination for infants only while adults are encouraged to be vaccinated (see S1 File). If the respondents answered “yes” to give bid amount indicate as 1; if answered “no” indicate as 0. The flowchart how the WTP was measured during the survey is presented in Fig 1.

#### Independent variables

We assessed three main groups of factors that would plausibly affect WTP: (1) price (bid amounts ranged between RM150 (US$36) and RM500 (US$120), in RM50 (US$12) increments, and were randomly given to respondents; (2) socio-demographic characteristics (gender, age, marital status, ethnicity, employment status, education level, and family income) and (3) perceptions. For statistical analysis purposes, Indian was collapsed with the “other” ethnicity, leaving three categories: Malay, Chinese, and other ethnicity. Education was dichotomized into those with a degree (having a degree or being a postgraduate) and those without a degree (i.e., no schooling, primary and secondary school and diploma). Seven types of occupation were assigned to classify the job of the participants: farmer, civil servant, private employee, self-employment, public sector, retired and other (included student and housewife). Family income was defined as the average income of members of household assessed used open ended question.

Several questions from the HBM (i.e. measured the perception domains) were included on the scale. There were three questions related to perceived susceptibility to HepB, four questions related to perceived severity (i.e., consequences of becoming infected with HBV), five questions related to perceived benefits of HepB vaccination, three questions related to perceived barriers to preventing HepB and cues to action for HepB vaccination. Each question was rated on a 7-point Likert scale from 1 (strongly disagree) to 7 (strongly agree), and responses for items within a domain were added together. Therefore, additive scale scores ranged from 3 to 15 for perceived susceptibility, perceived barriers and cues to action, 4 to 28 for perceived severity and 5 to 35 for perceived benefits.

### Statistical analysis

A logit regression model was estimated with explanatory variables that included socioeconomic status, perceptions, and the initial bid amount offered to the respondent. Three variables that were consistently found to be significant determinants of socioeconomic status in existing literature were entered into the logit regression as categorical variables: income [41–46]–entered as a continuous variable, education [41–44, 47–49]–entered as a dichotomous variable and those without a degree were the reference, and ethnicity [42, 48]–with the “other” category being the reference. MacFadden Pseudo R^2^ [50], predictive power regression and Hosmer-Lemeshow chi-square [51] were computed to evaluate model fit. A predictive ability of over 50% was deemed acceptable for a good model [52].

In this study, CVM was presented in a discrete choice econometric model to estimate the value of WTP for HepB vaccination. Hanemann [36] and Adamowicz *et al*. [53] have detailed specifications for WTP in this context. Based on Cameron’s formulation, [54], WTP was specified as: (Yes) = 1−{1+exp^V^}^−1^

Where P(Yes) is probability of yes responses, V is the monetary amount price of the self-paid HBV vaccination presented to respondents.

Based on the logit regression, the distribution of WTP for self-paid vaccination was obtained using equation:

$${P(Yes)=1-\{{1+exp}^{\left(\beta_{0}+\beta_{1}*{Bid}_{i}+{\beta_{2}*EDU}_{i}+\beta_{3}*{INC}_{i}+\beta_{4}*{MLY}_{i}+\beta_{5}*{CN}_{i}+\beta_{6}*{PS}_{i}+\varepsilon_{i}\right)}\}}^{-1}$$

The mean WTP for this study was estimated using coefficient value with significant variables as follows:

$$Mean\;WTP=[{(\beta }_{2}EDU+\beta_{3}INC+\beta_{4}MLY+\beta_{5}CN+\beta_{6}PS)/(\beta_{1})]$$

Where β_1_ = Coefficient for WTP bids; β_2_ = Coefficient for Education (Degree *vs*. non degree); β_3_ = Coefficient for Income; β_4_ = Coefficient for Ethnicity (Malay *vs*. others); β_5_ = Coefficient for Ethnicity (Chinese *vs*. others); and β_6_ = Coefficient for perceived susceptibility.

As a sensitivity analysis, two other models were constructed, one with just education and income, the second with education, income, and ethnicity. These models were discarded based on an overall consideration of model fit (results not shown). The difference in the estimated WTP values for sociodemographicstatus was analysed using the Mann-Whitney-U test. Price elasticity was calculated using the midpoint method [55]. Demand is elastic when the absolute value is more than 1, and inelastic when less than 1.

As an additional sensitivity check, we used Turnbull estimators, a non-parametric method, to estimate WTP [56]. Turnbull estimation used Stata 15.0, and all other statistical analyses were performed using SPSS v22 or NLogit 4 and Minitab 18. Significance was assessed at α = 0.05.

## Results

### Respondents’ characteristics

In this study, 768 households located in nine districts of Selangor state, Malaysia were recruited to participate. Among these, 40 were excluded due to non-response, unfinished interviews and incomplete or missing information, leaving a total of 728 (94.8%) observations with complete responses. The vast majority (60.3%) of respondents were Malay (Table 1), and most respondents had a higher education than secondary education (46.3%); few (1.7%) had never been to school. The mean monthly income of the household was RM4421 (US$1061), ranging from RM300 (US$72) to RM60000 (US$14438).

**Table 1:** Demographic distribution and perceptions among participants from Selangor, Malaysia, 2016 (N = 728).

Variable	Frequency (%)
Age (year)	40±11.0*
Age group (year)	
25–34	265 (36.4)
35–44	218 (29.9)
45–54	154 (21.2)
55 and above	91 (12.5)
Sex	
Male	397 (54.5)
Female	331 (45.5)
Ethnicity	
Malay	439 (60.3)
Chinese	170 (23.4)
Indian	116 (19.9)
Others	3 (0.4)
Occupation	
Civil servant	96 (13.2)
Private employee	214 (29.4)
Self-employment	175 (24.0)
Retired	53 (7.3)
Student	26 (3.6)
Others	19 (2.6)
Unemployed	145 (19.9)
Marital status	
Single	139 (19.1)
Married	574 (78.8)
Widowed	9 (1.2)
Divorced	6 (0.8)
Literacy	
Illiterate (never been to school)	13 (1.7)
Literate	715 (98.3)
Education	
Primary	36 (4.9)
Secondary	342 (47.1)
Diploma	188 (25.9)
Degree	123 (16.9)
Postgraduate	26 (3.6)
Monthly income (Ringgit Malaysia)	4421.21±3856*
Monthly income group (Ringgit Malaysia)	
≤2000	172 (23.6)
2001–3000	172 (23.6)
3001–4000	125 (17.2)
4001–5000	88 (12.1)
>5000	171 (23.5)
Perception of susceptibility (scale 3–15)	11.72±4.3*
Perception of severity (scale 4–28)	22.44±5.3*
Perception of benefit (scale 5–35)	28.67±5.5*
Perception of barrier (scale 3–15)	8.34±3.9*
Cues to action (scale 3–15)	16.42±4.0*

* Mean ± Standard deviation

### Willingness to pay for HepB vaccination

We found that 273 (37.5%) of respondents were willing to pay for HepB vaccination (Table 2). In this study the number of cumulative responses for each bid was different because we used a CMV survey with a single-bounded closed-ended dichotomous choice question in which each respondent was asked their WTP once using a random bid. The percentage of respondents who were willing to pay RM150 (US$36.1) was much higher compared to those who were willing to pay RM500 ($120), 67.0% *vs*. 21.1%. The mean and median WTP was RM303 (US$73).

**Table 2:** Distribution of willingness to pay for hepatitis B vaccination, Selangor, Malaysia, 2016.

WTP value	Willingness to pay				Cumulative frequency
	Yes		No		
	Frequency	%	Frequency	%	
RM150	67	67.0	33	33.0	100
RM200	36	40.9	52	59.1	88
RM250	31	37.3	52	62.7	83
RM300	33	36.3	58	63.7	91
RM350	37	39.8	56	60.2	93
RM400	20	20.8	76	79.2	96
RM450	33	32.7	68	67.3	101
RM500	16	21.1	60	78.9	76
Total	273	37.5	455	62.5	728

The estimated WTP was influenced significantly by gender, ethnicity, literacy and educational attainment (Table 3). The highest mean WTP was estimated for degree holders at RM222 (US$53) and the lowest estimated WTP was among those illiterate at RM45 (US$10). According to a non-parametric analysis, the mean WTP using Turnbull estimators was RM201 (variance RM103). The mean WTP in this method was between RM150 and RM200.

**Table 3:** Mean of willingness to pay according to demographic factors (N = 273).

Variable		Mean	
	N	WTP (RM)	P-value
Age group			
25–34	93	135.70	0.845
35–44	86	128.45	0.225
45–54	57	137.73	0.938
55 and above	37	159.00	0.068
Sex			
Male	156	146.91	0.017*
Female	117	123.79	0.017*
Ethnicity			
Malay	156	124.13	0.002*
Chinese	88	183.90	0.000*
Indian	29	63.90	0.000*
Occupation			
Civil servant	32	160.47	0.073
Private employee	92	147.89	0.104
Self-employment	73	126.85	0.199
Retired	11	172.77	0.125
Student	23	155.41	0.242
Others	36	90.60	<0.001**
Unemployed	6	110.67	0.409
Marital status			
Single	54	151.55	0.131
Married	213	133.56	0.175
Widowed	3	171.33	0.449
Divorced	3	84.83	0.250
Literacy			
Illiterate (never been to school)	3	45.50	0.044*
Literate	270	138.02	0.044*
Education			
Primary	14	119.18	0.386
Secondary	111	104.51	<0.001**
Diploma	66	97.55	<0.001**
Degree	63	222.41	<0.001**
Postgraduate	16	221.56	<0.001**
Monthly income group (RM)			
≤2000	52	105.48	0.001*
2001–3000	59	123.82	0.148
3001–4000	38	127.09	0.404
4001–5000	34	161.06	0.058
>5000	90	158.94	0.001*

* P<0.05

** P<0.001

### Factor associated with willingness to pay

In the initial stage of estimation, we included all variables in the logit regression model based on a priori considerations. The initial model suggested that age and gender influenced model fit negatively and therefore excluded. In the final multivariable logit regression model (Table 4), there was a negative relationship between bid amount and WTP for HepB vaccination: every one RM increase in the bid amount leads to 0.994 times as high of odds of being willing to pay (P<0.001). Family income, education, and family income were all significantly associated with WTP for HBV vaccination. Having a degree was associated with greater odds (2.708, P<0.001) of being willing to pay for HepB vaccination. Compared to other ethnicities, the odds of being willing to pay were 1.720 times greater for Malay and 2.968 times greater for Chinese.

**Table 4:** Factors associated with willingness to pay for hepatitis B vaccination, Selangor, Malaysia, 2016 (N = 728).

Variables	Coefficient (β)	Odds ratio	95% confidence interval	
			Lower	Upper
Constant	-1.142	0.319		
Bid	-0.006	0.994**	0.993	0.996
Education (Degree *vs*. no degree)	0.996	2.708**	1.772	4.137
Monthly income (in RM)	0.000	1.000**	1.000	1.000
Ethnicity (Malay *vs*. others)	0.542	1.720*	1.047	2.825
Ethnicity (Chinese *vs*. others)	1.088	2.968**	1.710	5.153
Perception of susceptibility	0.071	1.073**	1.032	1.116
Perception of severity	0.023	1.023	0.985	1.063
Perception of benefit	-0.019	0.981	0.947	1.017
Perception of barrier	0.005	1.005	0.962	1.050
Cues to action	0.024	1.025	0.975	1.077
Summary statistics				
Adopter correctly predicted	69%			
McFadden-R^2^	0.122			
Hosmer-Lemeshow Chi-square	5.921			
Number of observations	728			
Estimated mean WTP	RM303 (US$73) (95% CI:RM291-RM315)			
Estimated median WTP	RM303 (US$73) (95% CI:RM279-RM323)			
Standard deviation	101.25			
Standard error mean	6.13			
Number of observations	273			

* P<0.05

** P<0.001

Out of five domains of perception, only one domain, perceived susceptibility, was significantly associated with WTP for HepB vaccination. Greater perceived susceptibility to HBV infection was associated with 1.073 times greater odds of being willing to pay for HepB vaccination (P<0.001).

### Elasticity of demand

Self-paid HepB vaccination seems to be inelastic between RM150 (US$36) and RM350 (US$84) and the quantity demanded was less responsive to price changes, with price elasticity of -0.37 at RM150 and -0.92 at RM350 (Table 5). The quantity demanded appeared to be more price sensitive above RM400 (US$96).

**Table 5:** Price elasticity of demand.

Price for three doses HepB	Proportion willing to pay (%)	Price elasticity
RM150	55.5	-
RM200	50.0	-0.37
RM250	44.5	-0.52
RM300	39.2	-0.70
RM350	34.0	-0.92
RM400	29.3	-1.11
RM450	24.9	-1.38
RM500	21.0	-1.61

## Discussion

Malaysia has intermediate-high levels of HepB endemicity. Current government prevention methods have focused on vaccinating infants, but infection in adults remains a large problem and will likely continue to increase in incidence over the next decade [8]. In a cross-sectional study in Selangor, Malaysia, we found that respondents were willing to pay RM303 (US$73) for three doses of HepB vaccine. Three sociodemographic factors (educational attainment, ethnicity and family income), along with perceived susceptibility to HBV infection, were all associated with WTP for HepB vaccination.

Sociodemographic factors like educational attainment and ethnicity have commonly been found to be related to WTP in previous studies. In the context of WTP for interventions related to other infectious diseases, one study revealed a positive association between greater education and higher WTP [42], while others have found no consistent association [45, 57–60]. Moreover, our finding found that WTP was higher among Malay, and especially among Chinese, compared to others, is similar to previous studies which have found that ethnicity is significantly related to WTP in both the general population of a high-income country [61] and in low income areas [62].

Theoretically, when consumers consider paying for optional health services, their choices depend on their disposable income: greater income is positively associated with WTP [63]. Although one study on a hypothetical malaria vaccine in Nigeria found income to be negatively associated with WTP [64], most studies, for both infectious diseases [41, 45, 57] and non-infectious diseases [65–68] have been in concordance with this study, in that greater income or socioeconomic was associated with greater WTP.

Our study found that the mean WTP was higher than the prevailing market price for three doses of HepB vaccine. In fact, the vaccination coverage for HepB in Malaysia is still low. This indicates that behavioural (perception) domain factors are critical for someone to be vaccinated. Similar to past studies on HepB [69, 70] or HepB vaccination [15–17], our study used the HBM model as a framework for hypothesizing possible behavioural predictors of WTP. The modelling analysis of our HBM model from this study have been published elsewhere [18]. In the present study, only one component of this model, perceived susceptibility, was associated with WTP for HepB vaccination. In the United States, low perceived susceptibility was an important barrier to adolescent acceptance of the HepB vaccination [17]. In Korea, those who perceived themselves susceptible to human papillomavirus (HPV) were more accepting of the HPV vaccination [71]. In contrast, one study using a choice-based conjoint analysis to estimate European parents’ WTP for meningococcal conjugate vaccines showed that perceived risk was inconsistent with purchasing price [72]. However, our findings accord with most previous literature in that if individuals perceive their own susceptibility to HBV to be high, they would be more willing to pay for the HepB vaccination. Therefore, efforts to increase awareness of the disease and the vaccine is critical. One of the strategies to increase the WTP for HepB vaccination among inhabitants in Malaysia, especially in Selangor, would be to provide education about the susceptibility of individuals to HBV infection. Such strategies could include well designed information campaigns delivered thought mass media or social media. In addition, the government should consider conducting awareness programmes, focusing on individuals’ susceptibility to the disease, in higher learning institutions and communities with large populations of adults. In addition, specific programs such as forums, seminars and continuous education on preventive measures for HepB are still needed to reduce HBV transmission using non-vaccine measures. These programs could be conducted by government authorities of Malaysia.

Although previous studies found that perceived severity [73], perceived benefits [73], perceived barriers and cues to action cues to action [16, 73] were associated with health-related WTP, we did not find any relationship of these domains to WTP on HepB vaccination. Similarly, in a study in the neighbouring country of Singapore, there was no difference in perceived severity and susceptibility between chronic HepB patients with and without recent HepB screening [70]. These findings indicate that larger cultural factors inform which factors from models like the HBM are relevant within a particular population.

The demand for self-paid HepB vaccination in Malaysia was price inelastic at price below RM350 (US$84) and elastic in demand at price above RM400 (US$96). This study findings similar to the vaccine price elasticity for dengue, were the price inelastic at all price level except the highest price level with elastic demand [41]. Yet, price elasticity of demand for influenza in Japan shows that elastic in demand for rural area and inelastic demand for urban area [74].

This study has some limitations. Participants might tend to give favourable answers during the interview as a form of social desirability bias [75]; for example, if they perceive the vaccine to be a good thing, they may overestimate how much they are willing to pay for it. Hypothetical bias may have arisen in this study where participants misstate their actual preferences in a hypothetical survey compared to a real-life situation [59]. Additionally, we did not measure whether the participant already had been vaccinated, which could have impacted their response to a bid. This study however has some strengths. Households were selected randomly from a population-based sample. The WTP bid amounts were given to respondents randomly and this reduces the strategic bias that could arise when participants are asked to state a monetary value of WTP in open-ended questions. By using the closed-ended dichotomous choice method, we could estimate the true, unobservable value from ‘yes’ and ‘no’ responses in the various bid amounts [76]. Additionally, randomly assigning the bid amount for each respondent mitigates the potential for an anchoring effect bias [59].

## Conclusions

This study investigated WTP for HepB vaccination among Malaysians. On average, respondents were willing to pay RM303 (US$73) for HepB vaccination. Public awareness could be increased through programs such as public lectures at post-secondary institutions. Because ethnicity was also significant, brochures, awareness programmes, and public screenings on HepB could focus on specific communities, like Indians. Greater acceptance of HepB vaccination in the public could lead to greater acceptance of public funding mechanisms. Countering projected increases in the incidence of HepB disease in adults in Malaysia will require strategic planning to promote the vaccine, and will likely require campaigns to increase awareness of susceptibility to HBV infection or will require subsidies from the government to incentivize the public to vaccinate.

## Supporting information

S1 File Questionnaire used in the study. (PDF)
