# Supplementary material for: Willingness to pay for hepatitis B vaccination in Selangor, Malaysia: A cross-sectional household survey
Source: PLoS One. 2019 Apr 9;14(4):e0215125. doi: 10.1371/journal.pone.0215125 (PMC6456223; doi:10.1371/journal.pone.0215125)
Supplement: S1 File — (PDF) [file pone.0215125.s001.pdf]

Additional file 1 - Questionnaire used in the study

Demographic data

1. Age : ☐ Years

2. Gender
   - ☐ Male
   - ☐ Female

3. Race
   - ☐ Malay
   - ☐ Chinese
   - ☐ Indian
   - ☐ Others: ________________________

4. Marital status
   - ☐ Single
   - ☐ Married
   - ☐ Divorced
   - ☐ Widow/Widower

5. Occupation
   - ☐ Public sector
   - ☐ Private sector
   - ☐ S Self-employed
   - ☐ Student
   - ☐ Retired
   - ☐ Unemployment
   - ☐ Others: ________________________

6. Educational attainment
   - ☐ Never been to school
   - ☐ Primary school
   - ☐ Secondary school
   - ☐ Diploma/certificate
   - ☐ Undergraduate
   - ☐ Post graduate

7. Household monthly income: ☐ RM

Perceived Susceptibility

1- Strongly disagree
2- Disagree
3- Somewhat disagree
4- Neither agree or disagree
5- Somewhat agree
6- Agree
7- Strongly agree

No	Question							
1	I am less likely than most people to get infected with hepatitis B virus	1	2	3	4	5	6	7
2	My body could fight off hepatitis B virus infection.	1	2	3	4	5	6	7
3	I never worry about getting infected with hepatitis B virus	1	2	3	4	5	6	7

Perceived Severity

1- Strongly disagree
2- Disagree
3- Somewhat disagree
4- Neither agree or disagree
5- Somewhat agree
6- Agree
7- Strongly agree

No	Question							
1	I believe that I am at a higher risk of hepatitis B virus infection	1	2	3	4	5	6	7
2	I believe that my ethnic group is at a higher risk of hepatitis B virus infection	1	2	3	4	5	6	7
3	I belief that hepatitis B virus infection is a serious disease	1	2	3	4	5	6	7
4	I believe that HB infection leads to death	1	2	3	4	5	6	7

Perceived Benefit

1- Strongly disagree
2- Disagree
3- Somewhat disagree
4- Neither agree or disagree
5- Somewhat agree
6- Agree
7- Strongly agree

No	Question							
1	I believe if I get the hepatitis B vaccine, I shall be protected from hepatitis B virus infection	1	2	3	4	5	6	7
2	If I take the hepatitis B vaccine, it will reduce my worry about liver disease	1	2	3	4	5	6	7
3	I believe in the effectiveness of the hepatitis B vaccine now	1	2	3	4	5	6	7
4	I believe a vaccine for hepatitis B strengthens the immune system against hepatitis B virus	1	2	3	4	5	6	7
5	I believe that getting the hepatitis B vaccine is a good way to protect yourself from hepatitis B virus infection	1	2	3	4	5	6	7

Perceived Barriers

1- Strongly disagree
2- Disagree
3- Somewhat disagree
4- Neither agree or disagree
5- Somewhat agree
6- Agree
7- Strongly agree

No	Question							
1	I believe that the vaccination is not effective for me	1	2	3	4	5	6	7
2	I believe that the hepatitis B vaccination is likely to cause more harm than good	1	2	3	4	5	6	7
3	I do not have the time to get the vaccination	1	2	3	4	5	6	7

Cues to action

1- Strongly disagree
2- Disagree
3- Somewhat disagree
4- Neither agree or disagree
5- Somewhat agree
6- Agree
7- Strongly agree

No	Question							
1	I think the screening for HB infection is a good practice	1	2	3	4	5	6	7
2	An additional dose (booster) of the vaccine for hepatitis B should be taken when needed	1	2	3	4	5	6	7
3	I think all members of the family and friends should get the hepatitis B vaccine	1	2	3	4	5	6	7

Willingness to pay

The World Health Organization estimates that 240 million man has been infected with chronic hepatitis B virus in low and middle income countrie . In addition, there are 650,000 individuals die each year as a result of cirrhosis of the liver.

In Asia, it is estimated that more than 350 million people is a people with Hepatitis B and C. If the combined total of both Hepatitis sufferers, this became the ' killer ' the greatest man in the world.

Hepatitis briefly described as inflammation of the liver and is often caused by a virus. Hepatitis B is a virus that is capable symptoms of chronic and long term infection. About 30% of individuals who get hepatitis B infections in the long run tend to get complications such as liver cirrhosis or liver cancer.

Hepatitis briefly described as inflammation of the liver and is often caused by a virus. As we know, the heart is an organ in the top right of the stomach which has many functions, among them to save fuel for the body derived from sugar. In addition, to help the process of digestion of fats and proteins, an important protein for blood clots, began working on a variety of drug processing that you might take to be absorbed into bodily functions and help remove toxins from the body.

Viral Hepatitis B is spread through direct contact with infected blood or viruses of all kinds of bodily fluids. You will experience the signs of this disease within 45-160 days from the day you start infected. If infected, adults are more likely to suffer from symptoms of the disease than children. Hepatitis B can be prevented effectively through the Hepatitis B vaccine. In Malaysia, all babies are given a vaccination for the HBV by Government free of charge starting 1989. Hepatitis B vaccination for adults are also encouraged.

1. Based on the scenario above and your current income, you are willing to pay for a vaccine Hepatitis B if the cost of vaccines is **Bid Value(RM150, RM200, RM2050, RM300, RM350, RM400, RM450. RM500)** for three injections?
   ( ) Yes
   ( ) No

Data demografis

1. Umur : ☐ Tahun

2. Jantina
   - ☐ Lelaki
   - ☐ Perempuan

3. Keturunan
   - ☐ Malayu
   - ☐ Cina
   - ☐ India
   - ☐ Lain-lain: ________________________

4. Taraf perkahwinan:
   - ☐ Bujang
   - ☐ Kahwin
   - ☐ Bercerai
   - ☐ Janda/Duda

5. Occupation
   - ☐ Sektor awam
   - ☐ Sektor swasta
   - ☐ Bekerja sendiri
   - ☐ Pelajar
   - ☐ Bersara
   - ☐ Tidak bekerja
   - ☐ Lain-lain: ________________________

6. Pendidikan
   - ☐ Tidak pernah kesekolah
   - ☐ Sekolah Rendah
   - ☐ Sekolah Menengah
   - ☐ Diploma/Sijil
   - ☐ Sarjana Muda
   - ☐ Pascasiswazah

7. Berapakah pendapatan bulanan isirumah Anda: ☐ RM

Perceived Susceptibility

1- Sangat tidak setuju
2- Tidak bersetuju
3- Agak tidak setuju
4- Antara setuju dan tidak bersetuju
5- Agak setuju
6- Setuju
7- Sangat bersetuju

No	Soalan							
1	Peluang saya untuk dijangkiti dengan HepB adalah sangat sedikit jika dibandingkan dengan orang lain.	1	2	3	4	5	6	7
2	Badan saya boleh melawan jangkitan HepB.	1	2	3	4	5	6	7
3	Saya tidak pernah bimbang akan dijangkiti oleh HepB	1	2	3	4	5	6	7

Perceived Severity

1- Sangat tidak setuju
2- Tidak bersetuju
3- Agak tidak setuju
4- Antara setuju dan tidak bersetuju
5- Agak setuju
6- Setuju
7- Sangat bersetuju

No	Soalan							
1	Saya percaya bahawa saya berisiko tinggi untuk mendapat jangkitan HepB.	1	2	3	4	5	6	7
2	Saya percaya bahawa keturunan saya berisiko tinggi untuk jangkitan HepB.	1	2	3	4	5	6	7
3	Saya percaya bahawa jangkitan HepB adalah penyakit yang serius.	1	2	3	4	5	6	7
4	Saya percaya bahawa jangkitan HepB membawa kepada kematian.	1	2	3	4	5	6	7

Perceived Benefit

1- Sangat tidak setuju
2- Tidak bersetuju
3- Agak tidak setuju
4- Antara setuju dan tidak bersetuju
5- Agak setuju
6- Setuju
7- Sangat bersetuju

No	Soalan							
1	Saya percaya jika saya mendapat vaksin HepB, saya boleh dilindungi dari jangkitan HepB.	1	2	3	4	5	6	7
2	Jika saya mengambil vaksin HepB ia akan megurangkan kebimbangan saya mengenai penyakit hati	1	2	3	4	5	6	7
3	Saya percaya terhadap keberkesanan vaksin HepB sekarang.	1	2	3	4	5	6	7
4	Saya percaya vaksin HepB menguatkan system imun saya terhadap virus HepB.	1	2	3	4	5	6	7
5	Saya percaya bahawa mendapat vaksin HepB adalah cara yang baik untuk melindungi diri daripada jangkitan virus HepB	1	2	3	4	5	6	7

Perceived Barriers

1- Sangat tidak setuju
2- Tidak bersetuju
3- Agak tidak setuju
4- Antara setuju dan tidak bersetuju
5- Agak setuju
6- Setuju
7- Sangat bersetuju

No	Soalan							
1	Saya percaya bahawa vaksinasi tidak berkesan untuk saya.	1	2	3	4	5	6	7
2	Saya percaya bahawa vaksinasi HepB mendatangkan lebih banyak keburukan daripada kebaikan.	1	2	3	4	5	6	7
3	Saya tidak mempunyai masa untuk mendapatkan vaksinasi.	1	2	3	4	5	6	7

Cues to action

1- Sangat tidak setuju
2- Tidak bersetuju
3- Agak tidak setuju
4- Antara setuju dan tidak bersetuju
5- Agak setuju
6- Setuju
7- Sangat bersetuju

**No**	**Soalan**							
1	Saya rasa saringan untuk jangkitan HepB adalah amalan baik.	**1**	**2**	**3**	**4**	**5**	**6**	**7**
2	Dos tambahan vaksin HepB perlu diambil apabila diperlukan.	**1**	**2**	**3**	**4**	**5**	**6**	**7**
3	Saya rasa semua ahli keluarga dan rakan-rakan saya perlu mendapatkan vaksin HepB.	**1**	**2**	**3**	**4**	**5**	**6**	**7**

Willingness to pay

Pertubuhan Kesihatan Sedunia menganggarkan 240 juta manusia kronik telah dijangkiti oleh virus hepatitis B terutama di negara-negara berpendapatan rendah dan sederhana dan di global 240 juta individu sedang kronik dijangkiti dan 650,000 individu meninggal dunia setiap tahun akibat sirosis hati.

Di Asia, dianggarkan lebih daripada 350 juta penduduk merupakan penghidap Hepatitis B dan C. Jika dicampurkan jumlah penghidap keduanya, penyakit Hepatitis ini menjadi "pembunuh manusia" paling besar jumlahnya di dunia.

Hepatitis secara ringkasnya diterangkan sebagai keradangan hati dan selalunya dicetuskan oleh virus. Hepatitis B adalah virus yang mampu mencetuskan gejala kronik dan jangkitan jangka panjang. Kira-kira 30% dari individu yang mendapat jangkitan hepatitis B untuk jangka panjang akan cenderung mendapat komplikasi seperti sirosis hati atau kanser hati.

Sebagaimana yang kita tahu, hati adalah organ di bahagian kanan atas perut yang memiliki banyak fungsi, diantaranya menyimpan glikogen (bahan bakar untuk tubuh) yang diperoleh dari gula. Bila diperlukan, glikogen dipecahkan menjadi glukosa yang dilepaskan kedalam aliran darah.Selain itu, hati membantu proses pencernaan lemak dan protein, membuat protein yang penting bagi pembekuan darah, mengolah pelbagai ubat yang mungkin anda ambil untuk diserap ke dalam fungsi tubuh dan membantu membuang racun dari tubuh.

Virus hepatitis B tersebar menerusi perhubungan secara langsung dengan darah yang telah dijangkiti virus atau semua jenis cecair tubuh badan. Anda akan mengalami tanda-tanda penyakit ini dalam masa 45-160 hari dari hari anda mula dijangkiti. Jika dijangkiti, orang dewasa adalah lebih cenderung untuk mengalami tanda-tanda penyakit ini berbanding kanak-kanak. Hepatitis B boleh dicegah dengan efektif melalui vaksin Hepatitis B. Di Malaysia, semua bayi diberi vaksinasi untuk HBV oleh kerajaan secara percuma bermula tahun 1989. Vaksinasi Hepatitis B untuk golongan dewasa juga digalakkan.

1. Berdasarkan senario di atas dan pendapatan semasa anda, anda bersedia untuk membayar vaksin Hepatitis B, jika kos vaksin adalah **Bid value**(**RM150, RM200, RM2050, RM300, RM350, RM400, RM450. RM500)** untuk tiga suntikan?

( ) Ya
( ) Tidak
